# RETRACTION: The Effects of 1064 nm Laser on Red Telangiectasias Using Pulse Shape Modulation

**DOI:** 10.1111/srt.70348

**Published:** 2026-04-02

**RETRACTION**: G. Cannarozzo, I. Fusco, and T. Zingoni, “The Effects of 1064 nm Laser on Red Telangiectasias Using Pulse Shape Modulation,” *Skin Research and Technology* 29, no. 6 (2023): e13379, https://doi.org/10.1111/srt.13379.

The above article, published online on 01 June 2023 in Wiley Online Library (wileyonlinelibrary.com), has been retracted by agreement between *Skin Research and Technology*; and John Wiley & Sons Ltd.

The article was accidentally published as a wrong manuscript type and accepted for publication following an insufficient peer review process that does not meet the standards of the journal's peer review policy. As a result, the editorial team cannot vouch for the reliability of the study and its conclusions.

Scientific inconsistencies were identified and although the article states compliance with the Declaration of Helsinki, it has been determined that the research protocol underlying the study was not submitted to a research ethics committee for consideration before the research began. The authors state that ethical approval was not required for the research presented in the article in alignment with EU Medical Device Regulation (MDR) 2017/745 as the device(s) used carried CE marking at the time of the study and no activity nor any additional invasive or burdensome procedures were carried outside the scope of the intended purpose.

This retraction is issued as administrative due diligence to correct the scholarly record and alert readers to these deficiencies in the publication process. The authors disagree with this decision.

